# Matrix Regenerating Agent (RGTA) in a Neurotrophic Corneal Ulcer

**DOI:** 10.7759/cureus.11167

**Published:** 2020-10-26

**Authors:** Sara Pereira, Rui Resende, Pedro Coelho, Filipa Sampaio

**Affiliations:** 1 Ophthalmology, Hospital Pedro Hispano, Porto, PRT

**Keywords:** neurotrophic ulcer, matrix regenerating agent, cornea

## Abstract

Neurotrophic keratopathy is a condition associated with corneal damage and impaired corneal healing. There are no specific treatments available for this disease and current treatments are not associated with improved visual function. Matrix regenerating agents (RGTA) are recent topical agents showing positive results in the treatment of several corneal conditions, including neurotrophic keratopathy.

We report the case of a 73-year-old patient with a neurotrophic ulcer treated with RGTA.

Treatment with RGTA allowed complete corneal healing and a dramatic recovery in visual function in our patient. RGTA solutions are an important and safe therapeutic option for the treatment of selected corneal pathology.

## Introduction

Neurotrophic keratopathy is a rare degenerative corneal disease caused by trigeminal damage. This condition is characterized by a decrease in corneal sensitivity and impaired corneal healing. Currently, there are no specific medical treatment and management depends on the severity and stage of the disease. Some surgical approaches are useful in preserving eye integrity, however, these treatments do not show a significant impact on visual function [1].

Matrix regenerating agents (RGTA) are recent topical agents that show encouraging results in promoting tissue healing and regeneration, including cases of neurotrophic ulcers, corneal dystrophies or postsurgical conditions [2, 3].

## Case presentation

**Figure 1:**
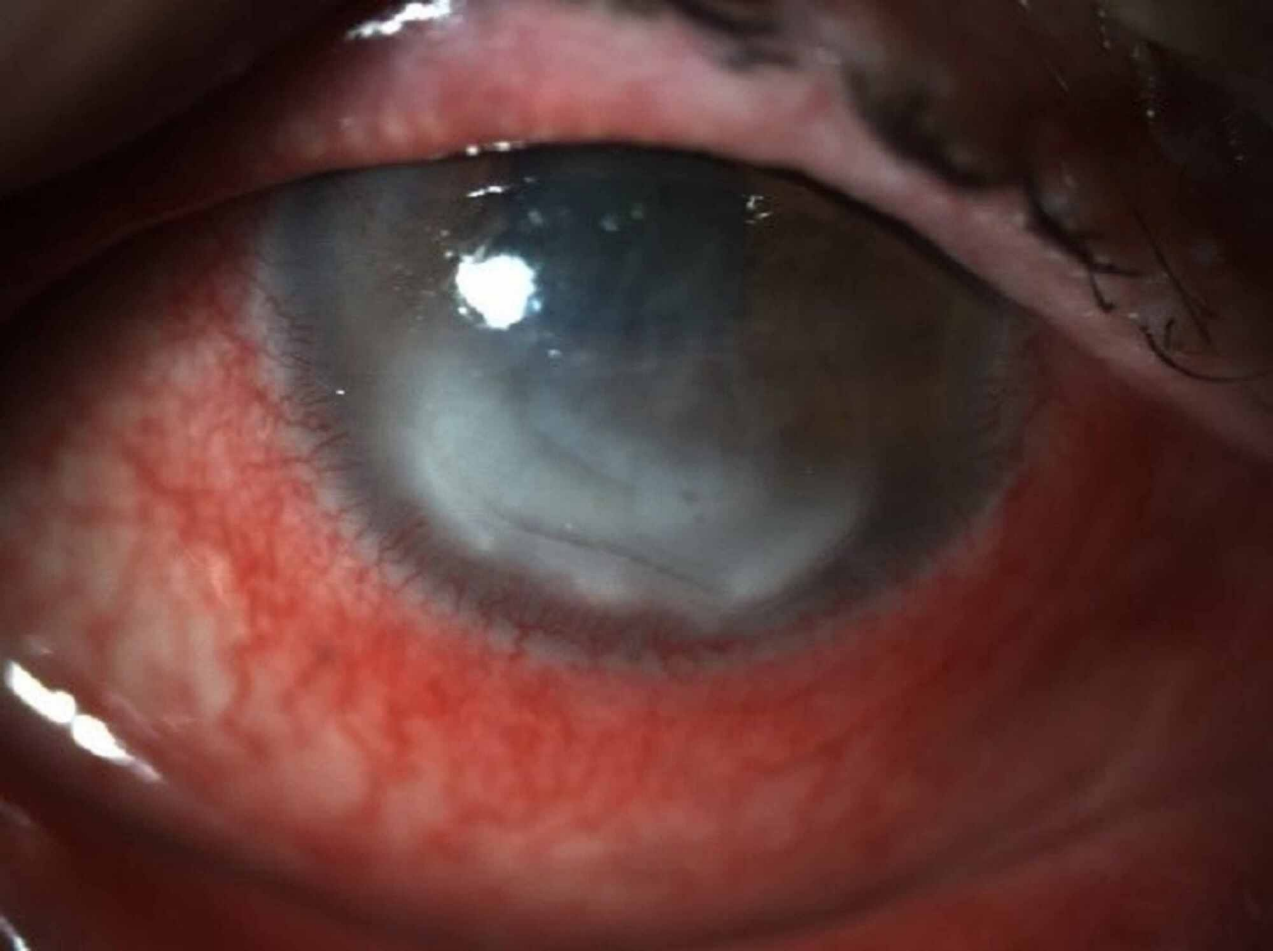
Corneal ulcer before treatment

A 73-year-old female patient presented to the emergency department complaining of ocular pain and decreased visual acuity on her right eye for about three weeks. Her medical records showed left eye amaurosis and arterial hypertension. On examination, she presented best corrected visual acuity of hand movements in the right eye, marked conjunctival injection and an extensive corneal ulcer occupying the inferior half of the eye (Figure 1).

**Figure 2:**
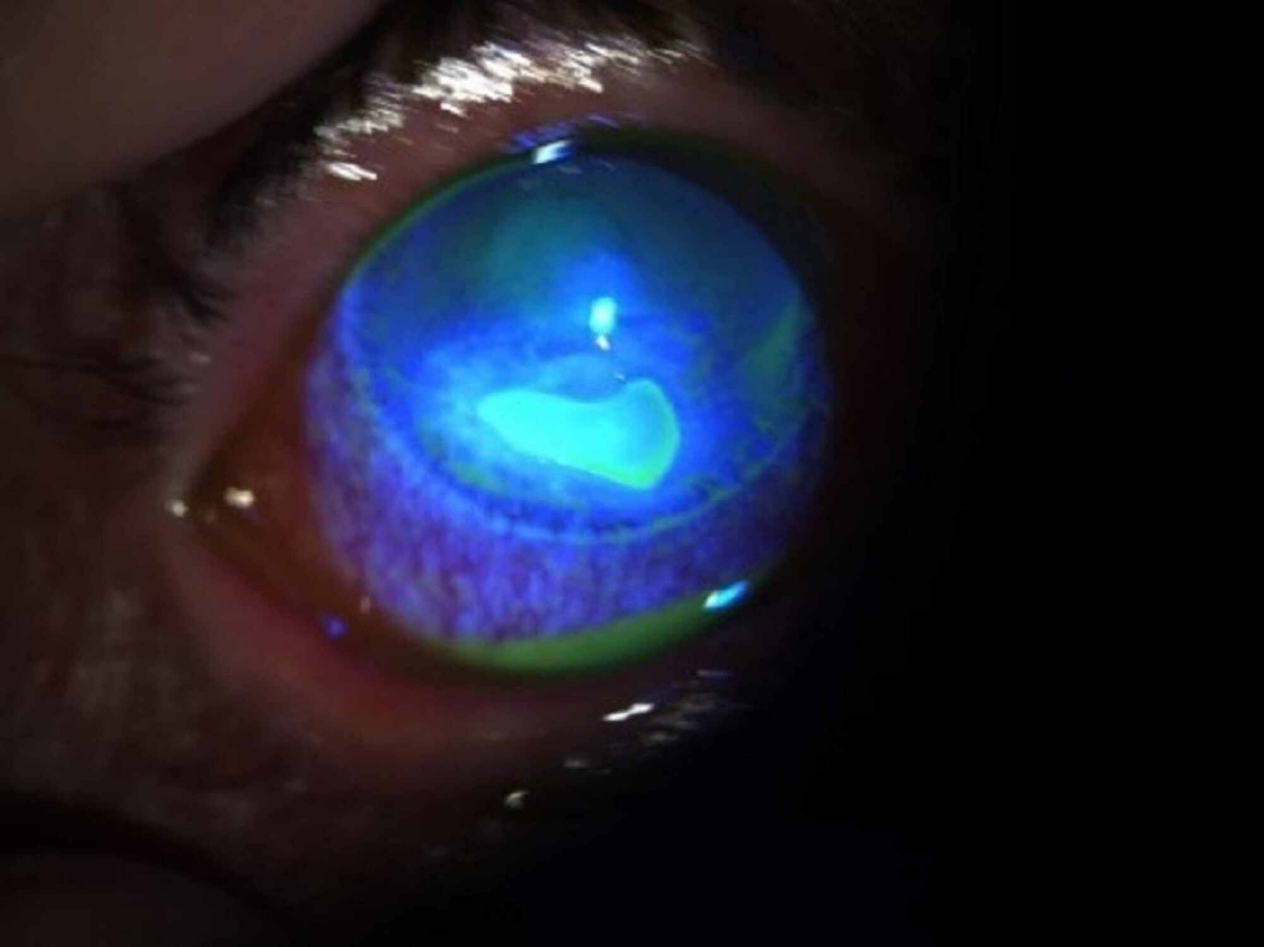
Corneal ulcer one week after treatment with antibiotics

The patient was started on topical broad-spectrum antibiotics, without significant improvement after one week of treatment. Microbiological and virologic analysis were negative (Figure 2).

**Figure 3:**
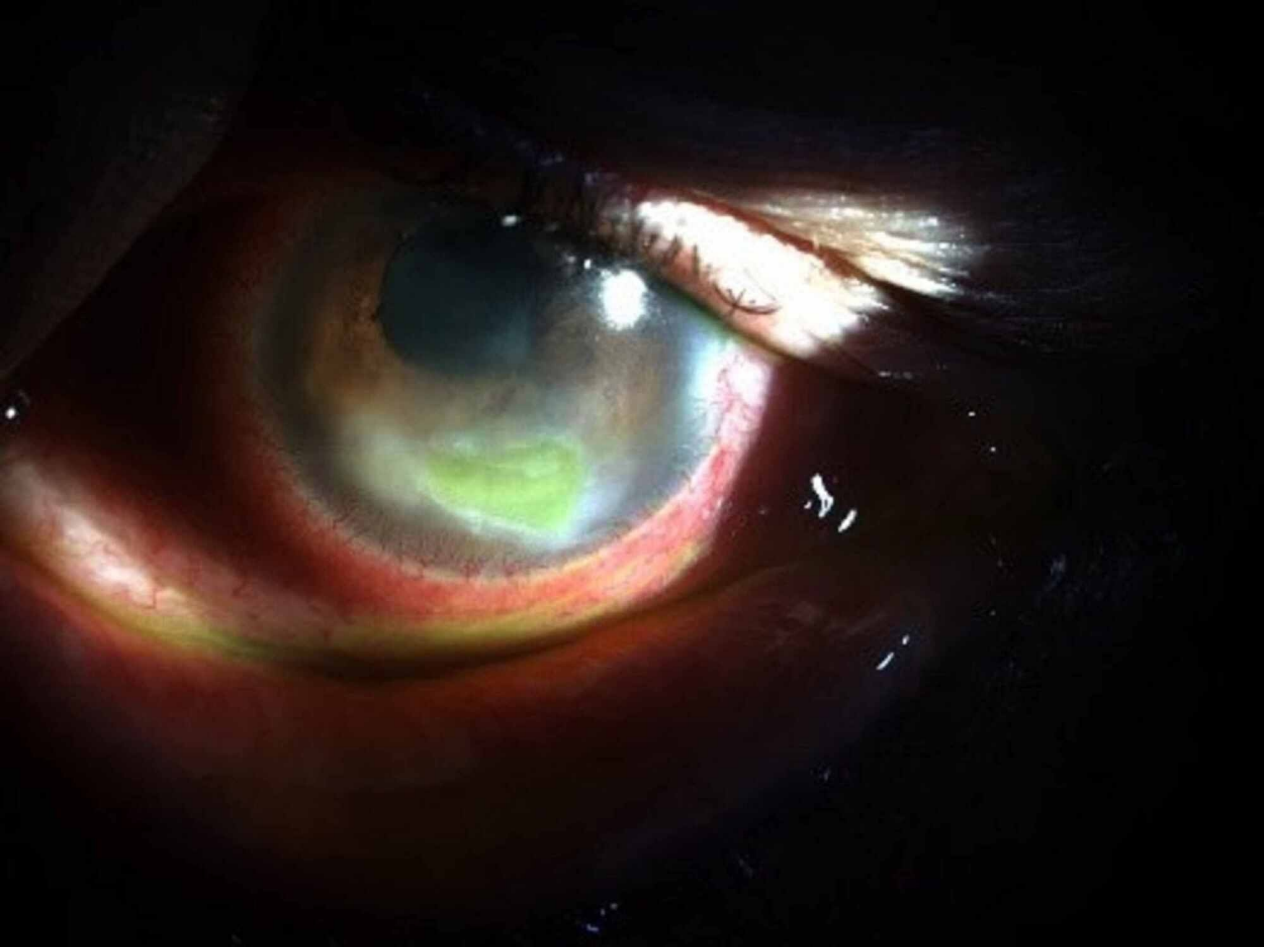
Corneal ulcer after three applications of RGTA

**Figure 4:**
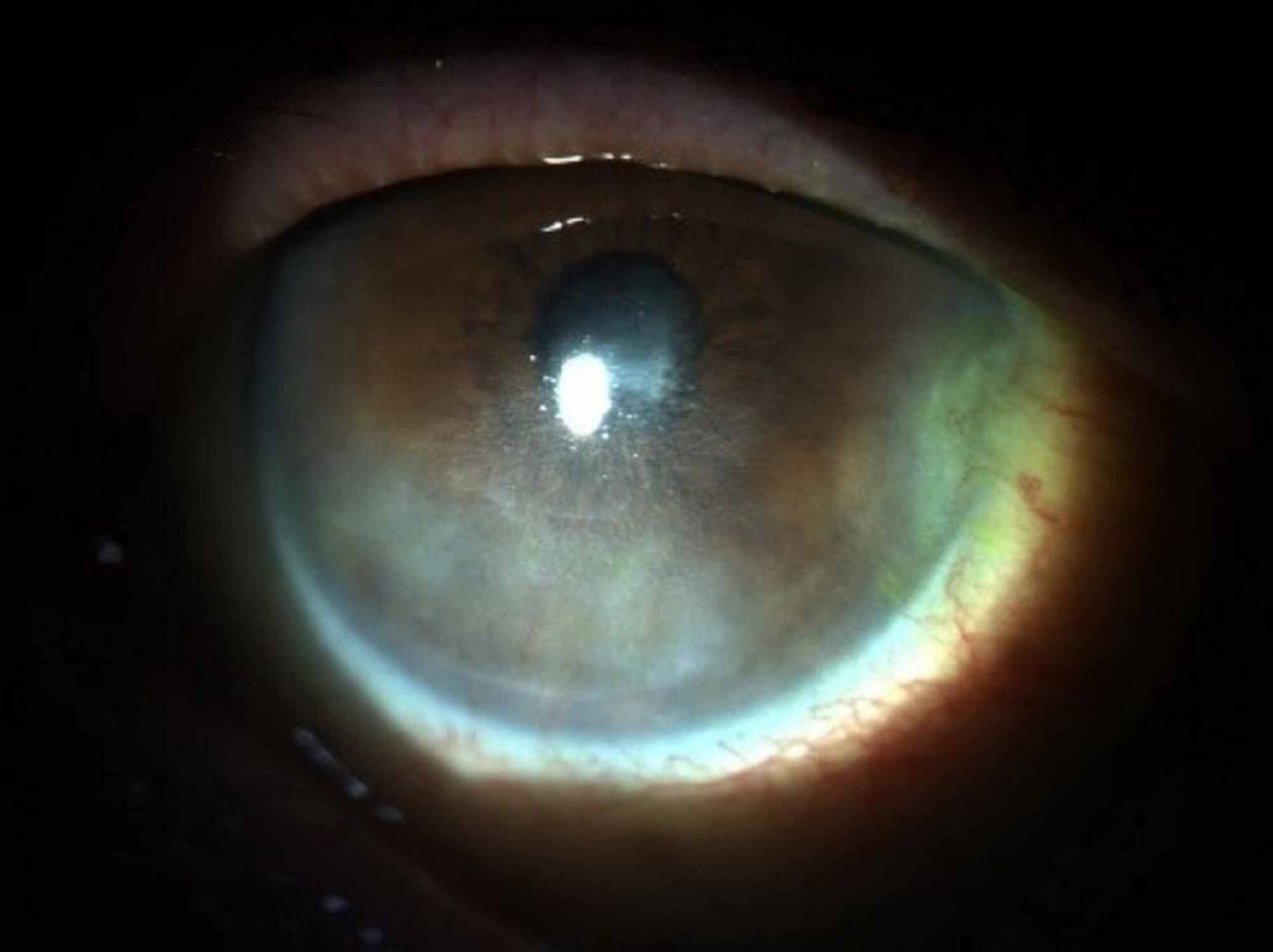
Corneal ulcer after 10 applications of RGTA

Given the possibility of a neurotrophic ulcer, the patient started application of RGTA eye drops - Cacicol ®, one drop every 48 hours. After 10 applications of RGTA - Cacicol ®, the patient showed complete ulcer healing with a significant decrease in ocular pain and surface inflammation and a marked improvement in best corrected visual acuity of 4/10 (Figures 3, 4).

## Discussion

Neurotrophic keratopathy is a rare entity characterized by impairment of corneal trigeminal innervation and development of corneal recurrent epithelial defects and ulceration. This disease can be potentially sight-threatening [1]. A neurotrophic ulcer is a diagnosis of exclusion and its treatment is a clinical challenge, with a specific treatment lacking.

Several management strategies are used for late stage neurotrophic ulcers, such as use of therapeutic contact lenses, amniotic membrane transplantation or surgical tarsorrhaphy and conjunctival flap. These procedures show variable rates of efficacy and safety and some of them present significant side effects [1, 3].

Regenerating matrix agent (RGTA), mimicking heparan sulfate, promotes regeneration of damaged tissues and enhances tissue healing. Several studies have demonstrated the efficacy of these agents on corneal healing, including not only neurotrophic ulcers, but also in re-epithelialization after penetrating keratoplasty and chronic corneal dystrophies [2-7]. This topical and preservative-free agent is very well-tolerated with the benefits of having a very simple application regimen and the potential to decrease possible surgical interventions.

The utility of RGTA agents is not limited to the ophthalmological field, with evidence of efficacy in the treatment of a wide spectrum of conditions, such as chronic skin and diabetic ulcers, oral and gastrointestinal tract associated lesions and tendon and muscle regeneration [8]. Its efficacy in wound healing and reducing fibrosis has made it useful in reconstructive surgical procedures and postoperative care [9].

In our case, the patient showed complete corneal reepithelialization with a profound recovery of visual acuity in a monocular patient with only three weeks of treatment. There was no treatment-related local or systemic side effects and the patient did not report pain or discomfort during application.

## Conclusions

Treatment with RGTA showed significant enhancement of corneal reepithelialization and a complete resolution of ocular inflammation.

RGTA ophthalmic solutions are an important and safe therapeutic option for controlling ocular surface inflammation and promoting corneal healing.
